# Utilization Patterns and Optimization Suggestions for Wildlife Passages in Xinjiang Nature Reserves

**DOI:** 10.1002/ece3.70969

**Published:** 2025-03-09

**Authors:** Mengdi Fu, Jun Wang, Shuang Li, Le Qin, Junsheng Li, Shichao Jin

**Affiliations:** ^1^ State Key Laboratory of Environmental Criteria and Risk Assessment, State Environmental Protection Key Laboratory of Regional Eco‐Process and Function Assessment Chinese Research Academy of Environmental Sciences Beijing China; ^2^ Center for Biodiversity and Nature Reserve Chinese Academy of Environmental Planning Beijing China; ^3^ Command Center for Comprehensive Survey of Natural Resources China Geological Survey Bureau Beijing China

**Keywords:** fence, influencing factor, linear infrastructure, utilization rate, wildlife passage

## Abstract

The expansion of linear infrastructure presents a significant threat to biodiversity, emphasizing the urgent need for regional studies on spatial variations and comprehensive multispecies research. This study surveyed and monitored wildlife passages across eight nature reserves in Xinjiang, evaluating their construction status, utilization patterns, and key factors influencing utilization rates, as well as providing optimization recommendations. The findings revealed that dedicated wildlife passages were scarce, especially in smaller reserves, which primarily relied on small bridges and culverts originally designed for water flow. Enhancing the construction of passages in these areas is strongly recommended. A total of 32 wildlife species were recorded, comprising 13 bird species and 19 mammal species. Ungulates, including bharal ( *Pseudois nayaur* ), goitered gazelle ( *Gazella subgutturosa* ), Asian wild ass ( *Equus hemionus* ), and wild Bactrian camel ( *Camelus ferus* ), exhibited high relative abundance indices, indicating a strong preference for utilizing passages. Passage utilization rates exhibited significant seasonal and diurnal variations. Winter usage was the lowest, followed by an increase in spring, peaking in summer and autumn. Birds exhibited particularly high passage utilization rates during migratory seasons, with the peak occurring in April. Additionally, nocturnal passage utilization rates were significantly higher than during other periods, with both sunrise and sunset showing positive selection, as indicated by 86.86% and 91.30% of monitored sites recording JSI > 0. To enhance the effectiveness of passages, seasonal and diurnal variations should be fully considered in passage construction and management, particularly by minimizing human activity during nighttime, dawn, and dusk. Utilization rates were significantly constrained by the intensity of human activity and the density of linear infrastructure. Passage type, size, and proximity to water were also critical factors. Optimizing passage layout, implementing dynamic management in grassland fence areas, increasing passage density, and enhancing ecological functionality are recommended strategies to facilitate wildlife movement and support biodiversity conservation.

## Graphical abstract

Linear infrastructure expansion poses a significant threat to biodiversity. We surveyed and monitored wildlife passages in eight Xinjiang nature reserves, assessing construction status, utilization patterns, and influencing factors. This study provides conservation strategies to improve wildlife passage construction and management, ensuring effective biodiversity protection.

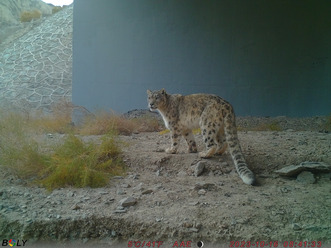

## 1 Introduction

The expansion of linear infrastructure, such as roads and fences, has significantly contributed to the loss and fragmentation of wildlife habitats (Underhill and Angold 1999; Trombulak and Frissell 2000; Geneletti 2004; Wang et al. 2022). This has adversely impacted wildlife migration and dispersal, increasing the risk of injury or mortality on roads (Coffin 2007; Glista, DeVault, and DeWoody 2009; Gunson, Mountrakis, and Quackenbush 2011). Consequently, it has emerged as one of the major threats to biodiversity (Forman and Alexander 1998; Ree et al. 2011). Constructing wildlife passages can mitigate the adverse effects of linear infrastructure on wildlife.

Research on wildlife passage utilization has primarily focused on their types and designs, including structural features (Clevenger, Chruszcz, and Gunson 2001; Dodd Jr., Barichivich, and Smith 2004; Rytwinski et al. 2015), vegetation cover (Martinig and Bélanger‐Smith 2016; Baechli, Albanesi, and Bellis 2021), and fencing (McCollister and van Manen 2010). For instance, studies have shown that large mammals generally prefer wide passages with dense vegetation cover (Jackson and Griffin 2000), whereas small mammals exhibit less stringent requirements (Wang et al. 2018). The effectiveness of passages is typically assessed by monitoring animal crossing frequencies and success rates, alongside observations of animal behavior near the passages (Seidler, Green, and Beckmann 2018; Mysłajek et al. 2020). However, the effectiveness of these passages is influenced by various factors, such as traffic volume (Jacobson et al. 2016), human activities near passages (Barrueto, Ford, and Clevenger 2014), and seasonal variations (Gagnon et al. 2011).

While progress has been made in studying the types, designs, and effectiveness of wildlife passages, substantial gaps persist. Most existing research focuses on specific regions, such as individual protected areas (Clevenger and Waltho 2000; Bakaloudis, Bontzorlos, and Kotsonas 2023) or major roads (Xia et al. 2007; Wang et al. 2017). Few comparative studies examine passage usage across different regions or varying types of linear infrastructure. In areas with high biodiversity and diverse ecosystems, understanding the spatial heterogeneity of passage usage patterns is crucial. Such studies not only reveal how wildlife adapt to different environments and infrastructures but also provide critical insights for optimizing passage design and regional biodiversity conservation strategies. Additionally, while some studies have focused on specific species (Gloyne and Clevenger 2001; Xu et al. 2019) or groups (Mata et al. 2008; Collinson et al. 2019; Mulualem et al. 2023), comprehensive studies on the usage of wildlife passages by multiple species remain scarce (Ng et al. 2004; Ważna et al. 2020; Baechli, Albanesi, and Bellis 2021). This is especially true in China's biodiversity conservation priority areas and key ecological functional zones, where systematic research on multispecies passage usage patterns remains virtually nonexistent. Given the significant differences in ecological requirements across species, comprehensive multispecies studies are essential for developing passages that are both widely applicable and capable of meeting diverse ecological needs.

The Xinjiang Uygur Autonomous Region (Xinjiang), characterized by diverse terrain and climates, supports a rich diversity of wildlife. However, it also features an expanding transportation network comprising highways, railways, and county‐level roads, as well as fences delineating grassland ownership, ecological protection zones, and road protection areas. The intersection of these linear infrastructures with nature reserves creates significant challenges for wildlife habitats and migration patterns (Zhang et al. 2022; Yang et al. 2024).

This study investigates wildlife passage usage patterns in representative nature reserves in Xinjiang, aiming to provide evidence‐based recommendations for optimizing passage construction and management. The specific objectives are as follows: (1) To investigate the current state of wildlife passage construction, including their spatial layout and structural characteristics; (2) To analyze species composition and their spatiotemporal distribution patterns using data from infrared cameras; (3) To examine passage usage patterns, focusing on seasonal and diurnal variations; (4) To identify key factors influencing passage utilization rates; and (5) To propose targeted recommendations to optimize passage construction. The innovation of this study lies in its multispecies integrated approach combined with spatial heterogeneity analysis, offering scientific insights for optimizing wildlife passages in biodiversity conservation priority areas and key ecological functional zones. Furthermore, the study hypothesizes that significant differences in passage usage exist among species, influenced by factors such as ecological habits, passage characteristics, and environmental disturbances.

## 2 Materials and Methods

### 2.1 Study Area

The Xinjiang Uygur Autonomous Region, located in the hinterland of the Eurasian continent, spans 15° of latitude and 23° of longitude, covering an area of 1,664,900 km^2^, making it the largest province in China. Xinjiang's climate exhibits significant variation between its northern and southern regions. The northern region experiences a temperate continental climate, while the southern region has a warm temperate continental climate, marked by extreme temperature fluctuations. The lowest temperature recorded in Fuyun County, located in northern Xinjiang, was −50.15°C, while the highest temperature recorded in Turpan City, in southern Xinjiang, was 49.6°C. Precipitation distribution also varies considerably, with the lowest annual precipitation recorded in Toksun County (8.1 mm) and the highest in Tianchi (571.7 mm). The lowest point is Ayding Lake, situated 155 m below sea level, while the highest point is Chogori Peak, rising 8611 m above sea level. The diverse landforms and climates of Xinjiang support a rich array of species, including 733 vertebrate species and over 10,000 invertebrate species. In total, 178 species are listed as key protected wildlife in China.

Xinjiang is home to three of China's priority biodiversity conservation areas: the Altai Mountains, the Tianshan Mountains–Southwest Junggar Basin, and the Tarim River Basin, as well as two key ecological function zones: the eastern and western biodiversity conservation areas of the Junggar Basin. As of now, Xinjiang has established 28 nature reserves, covering 200,200 km^2^, which accounts for 12% of the total land area. These reserves play a vital role in maintaining biodiversity and ecosystem functions. Within these reserves, the total length of roads and railways spans 15,200 km, and fences extend over 2800 km.

### 2.2 Survey and Monitoring Sites

**FIGURE 1:**
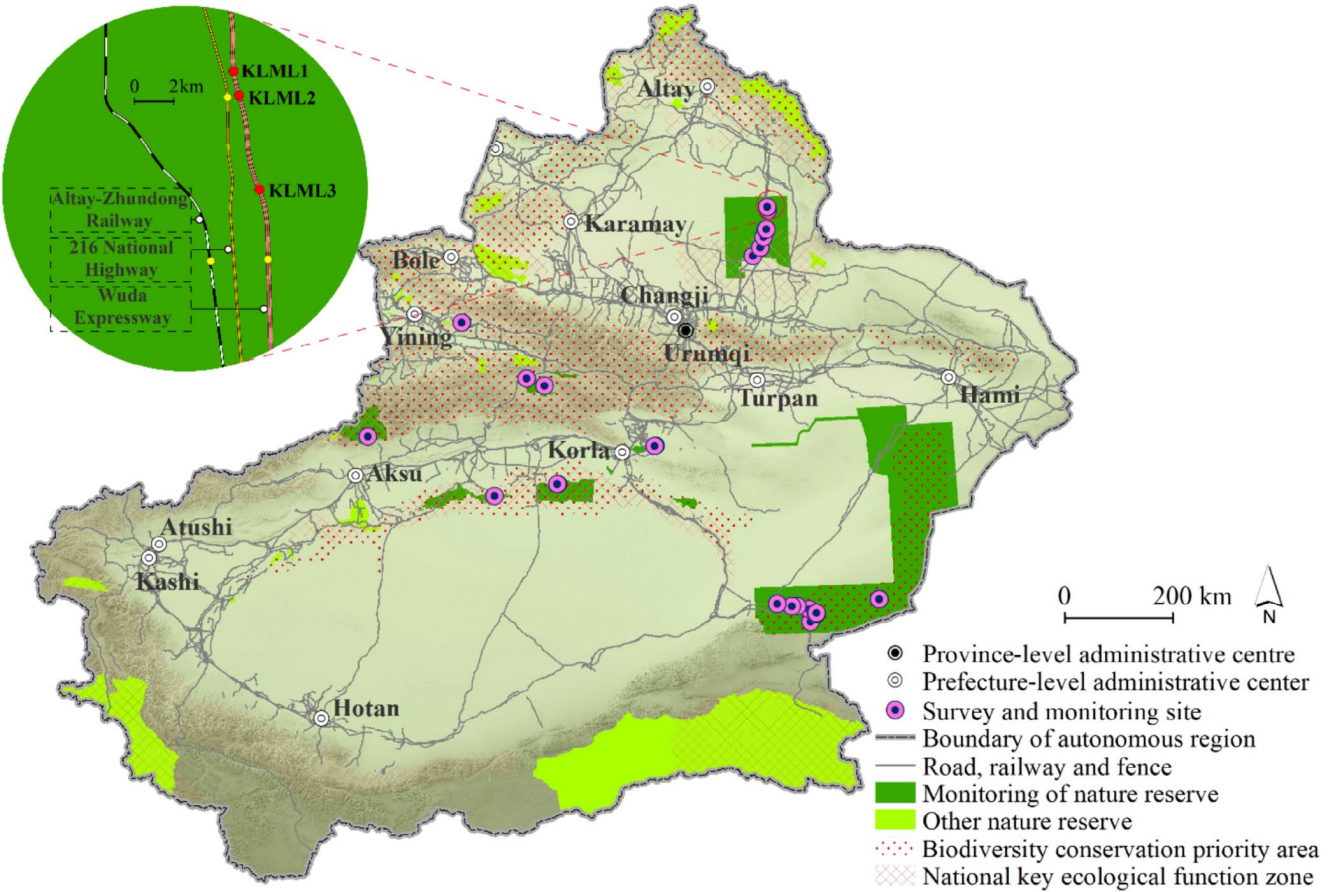
Spatial distribution of wildlife passage survey and monitoring sites.

This study selected eight nature reserves characterized by relatively high‐density linear infrastructure (exceeding 0.05 km/km^2^), located within China's biodiversity conservation priority areas and key ecological functional zones. These reserves account for 28.57% of the total number of nature reserves in Xinjiang and 43.85% of the total area of these reserves (Figure 1). Wildlife passage surveys and wildlife activity monitoring were conducted in these areas, with a focus on regions characterized by high concentrations of animal paths, bedding sites, and footprints.

**FIGURE 2:**
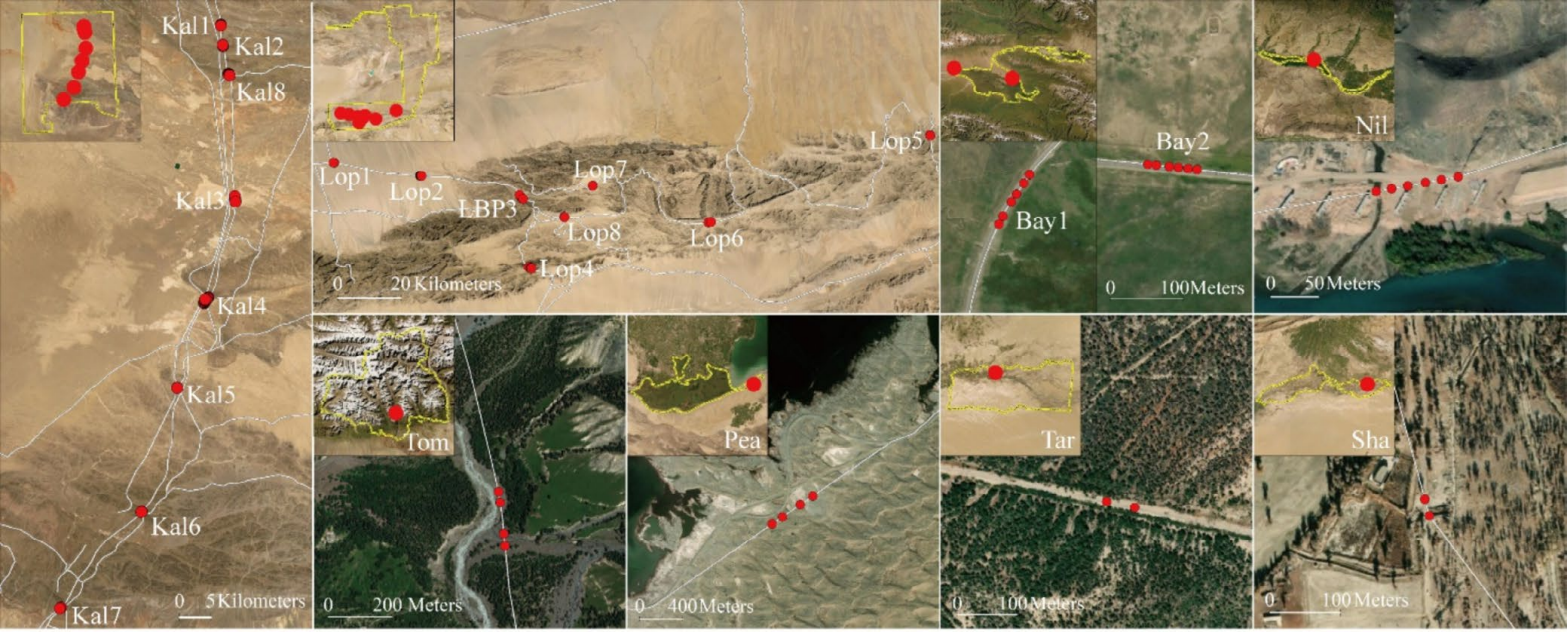
Spatial distribution of wildlife passage monitoring points. Bay, Bayingbrook National Nature Reserve; Kal, Kalamaili Mountain Ungulate Wildlife National Nature Reserve; Lop, Lop Nur Wild Bactrian Camel National Nature Reserve; Nil, Dense‐Leaved Poplar County‐Level Nature Reserve in the Nilekkashgar River Basin; Pea, Peacock River Wetland Prefecture‐level Nature Reserve; Sha, Autonomous Region‐level Wetland Nature Reserve in the Upper Reaches of the Tarim River in Shaya County; Tar, Tarim Populus Euphratica National Nature Reserve; Tom, Tomur Peak National Nature Reserve.

Monitoring points were established using stratified and systematic sampling methods (Tong 2006). In the stratified sampling method, monitoring points were distributed across different habitat types within the nature reserves. For each habitat type, one to three replicated monitoring points were established, with a minimum spacing of 500 m between points. In the systematic sampling method, monitoring points were established at 1 km intervals where linear infrastructure intersected areas of wildlife activity. Each monitoring site was assigned a unique code, and information including location, latitude and longitude, elevation, functional area, linear infrastructure type, passage type, and habitat type was systematically recorded. A total of 24 sites were selected, and 160 monitoring points were established across eight nature reserves. Due to the loss or damage of infrared cameras, 132 effective cameras were operational across 23 sample sites (Table 1, Figure 2).

**TABLE 1:** Deployment of survey and monitoring sites for wildlife passages.

Name of nature reserve	Major habitat type	Linear infrastructure type and density (km/km^2^)	Site code and number of monitoring points	Functional area	Passage type and size
Bayingbrook National Nature Reserve	Grassland	Road, 0.05	Bay1, 6	Experimental zone	Underpass, deck width 9 m, clearance height 2.6 m, span 16 m
			Bay2, 6	Buffer zone	Underpass, deck width 10 m, clearance height 4 m, span 40 m
Kalamaili Mountain Ungulate Wildlife National Nature Reserve	Desert	Road and railway, 0.08	Kal1, 10	Experimental zone	Underpass, deck width 21 m, clearance height 5.4 m, span 150 m
			Kal2, 14		Underpass, deck width 21 m, clearance height 5.4 m, span 360 m
			Kal, 12		Underpass, deck width 21 m, clearance height 5.4 m, span 260 m
			Kal4, 14		Underpass, deck width 21 m, clearance height 4 m, span 300 m
			Kal5, 2		Underpass, deck width 20 m, clearance height 2 m, span 3 m
			Kal6, 2		Underpass, deck width 20 m, clearance height 2 m, span 3 m
			Kal7, 6		Underpass, deck width 20 m, clearance height 2 m, span 15 m
			Kal8, 6		Overpass, deck width 48 m, clearance height 6 m, span 33 m
Peacock River Wetland Prefecture‐level Nature Reserve	Wetland	Fence, 0.13	Pea, 2	Experimental zone	Overpass, width 3 m
Lop Nur Wild Bactrian Camel National Nature Reserve	Desert	Road, railway and fence, 0.05	Lop1, 2	Experimental zone	Underpass, deck width 18 m, clearance height 2.4 m, span 4 m
			Lop2, 6		Underpass, deck width 7 m, clearance height 4.5 m, span 40 m
			Lop3, 6		Underpass, deck width 13 m, clearance height 3.3 m, span 22 m
			Lop4, 16		Underpass, deck width 28 m, clearance height 10 m, span 500 m
			Lop5, 2	Buffer zone	Overpass, width 5 m
			Lop6, 2	Experimental zone	Overpass, width 5 m
			Lop7, 2		Overpass, width 5 m
			Lop8, 2		Overpass, width 5 m
Dense‐leaved Poplar County‐Level Nature Reserve in the Nilekkashgar River Basin	Wetland	Road, 0.15	Nil, 6	Core zone	Underpass, deck width 12 m, clearance height 4 m, span 46 m
Autonomous Region‐level Wetland Nature Reserve in the Upper Reaches of the Tarim River in Shaya County	Farmland	Fence, 0.17	Sha, 2	Core zone	Overpass, width 3 m
Tarim Populus Euphratica National Nature Reserve	Forest	Road, 0.07	Tar, 2	Core zone	Overpass, width 3 m
Tomur Peak National Nature Reserve	Forest	Road, 0.13	Tom, 4	Core zone	Underpass, deck width 11 m, clearance height 2.5 m, span 6.7 m

### 2.3 Survey and Monitoring Methods

Discussions with local forestry and grassland bureaus, as well as nature reserve management institutions, provided information on key wildlife distribution and movement corridors. Field surveys documented wildlife passage types, bridge clearances, spans, heights, and habitats on both sides. Infrared camera monitoring technology, widely used in wildlife passage utilization studies (Cilulko et al. 2013), was employed in this study. Cameras were positioned 0.5 to 1 m above the ground, with a slight downward angle to avoid direct sunlight and reflective surfaces. The performance and settings of the cameras are provided in Appendix A: Table A1. Data were collected for at least 3 months at each monitoring point to account for variations in animal activity and ensure comprehensive data collection (Ng et al. 2004; Malo et al. 2005).

### 2.4 Data Sources and Processing

Species identification was based on the “List of Biological Species of China (2023 Edition),” the “Vertebrate Volume of the Red List of Biodiversity of China,” and the “List of Birds of China (4th Edition, 2023).” Photos containing clearly identifiable species were used to record species names and numbers. If a photo contained multiple species, it was divided into separate records for each species. Only independent valid photos were counted, defined as photos (or groups) of different individuals or groups of the same species taken at a monitoring point within 30 min (Kühl et al. 2023).

The key factors considered in influencing wildlife passage utilization rates included: vegetation cover, which affects concealment and food resources; the adaptability of passage types for different wildlife species; habitat types on both sides and the distance to water, which determine wildlife willingness to use the passage; the width and height of the passage, which determine the ability of animals of varying sizes to pass through; the density of linear infrastructure, which affects wildlife range and passage effectiveness; and traffic volume and human activity intensity, which directly affect wildlife passage usage. Predator pressure and food resource abundance, which are difficult to quantify and directly monitor, were not considered. The explanations of the data and their sources are provided in Table 2.

**TABLE 2:** Data explanation and sources.

Data category	Data description	Data source
Nature reserve vector data	According to the protection intensity of nature reserves (Hull et al. 2011; Xu et al. 2016), the core area, buffer zone, and experimental zone were assigned values of 3, 2, and 1, respectively. The higher the protection intensity, the higher the assigned value.	National Forestry and Grassland Administration: https://www.forestry.gov.cn/
Vector data of priority areas for biodiversity conservation in China	To implement the “China Biodiversity Conservation Strategy and Action Plan (2011–2030)” and enhance the protection and regulation of biodiversity priority areas, the Ministry of Ecology and Environment organized the delineation of boundaries for biodiversity priority areas, establishing the scope of China's biodiversity conservation priority areas.	Ministry of Ecology and Environment: https://www.mee.gov.cn/
Vector Data of China's Key Ecological Function Zones	Key ecological function zones, delineated by the state, serve as critical ecological security barriers. These zones are essential for soil and water conservation, water resource protection, wind erosion control, and biodiversity maintenance.	
Linear infrastructure vector data	Using remote sensing imagery, the spatial distributions of roads and railways were identified (Ramita, Odeh, and Tiho 2009), alongside the distribution of fences (Sluijs et al. 2020).	GF‐2 satellite: http://www.gscloud.cn
Vegetation coverage	The average vegetation cover within a 1 km radius around each sample site was calculated using remote sensing imagery.	
Distance from water	The straight‐line distance from each passage to the nearest permanent or seasonal water source was calculated. The spatial distribution of water sources was derived from remote sensing imagery.	
Traffic volume	It referred to the annual average traffic volume of motor vehicles, measured in vehicles per day.	Xinjiang Uygur Autonomous Region Department of Transportation: https://jtyst.xinjiang.gov.cn/xjjtysj/
Passage type	Underpass and overpass types of passages were assigned values of 1 and 2, respectively.	Fieldwork
Passage size	It referred to the volume of the passage (deck width of the bridge × clearance height × span) and the width of the passage reserved in the fence.	
Habitat type	The five types of habitats—desert, grassland, forest, farmland, and wetland—were assigned values of 1, 2, 3, 4, and 5, respectively.	
Linear infrastructure density	It referred to the ratio of the total length of highways, railways, and fences within a 1 km radius of the sample sites to the area of that region.	
Human activity intensity	It referred to the total number of independent valid photos of humans and livestock within the sample site divided by the total number of effective working days at all monitoring points.	

### 2.5 Data Analysis

Data mining employed indices such as the relative abundance index (Campbell et al. 2015), the Shannon–Wiener index (Strong 2016), and the Jacobs selection index (Jacobs 1974), calculated as follows:

$$\mathrm{RAI}=\frac{N_{i}}{D}\times 100$$

where RAI represents the relative abundance index, *N*_*i*_ is the number of independent valid photos of species *i* at the site, and *D* refers to the number of effective working days across all monitoring points at the site. A higher RAI indicates a greater abundance of species *i*.

$$\mathrm{SWI}=-\sum_{i=1}^{S}P_{i}\ln P_{i}$$

where SWI represents the Shannon–Wiener index, *P*_*i*_ is the proportion of independent valid photos of species *i* relative to the total number of independent valid photos, and *S* denotes the total number of species. A higher SWI indicates greater species diversity.

$${\mathrm{JSI}}_{i}=\frac{\left(r_{i}-p_{i}\right)}{r_{i}+p_{i}-2r_{i}p_{i}}$$

where *JSI*_*i*_ represents the Jacobs selection index for time period *i*, *r*_*i*_ is the passage utilization frequency of passages by wildlife during time period *i*, and *p*_*i*_ is the proportion of time period *i* relative to the total daily duration. JSI > 0 indicates positive selection, and JSI ≤ 0 indicates negative selection. The day was divided into six periods: night (1 h after sunset to 1 h before sunrise), sunrise (1 h before and after sunrise), morning (1 h after sunrise to 13:30), noon (13:30 to 16:30), afternoon (16:30 to 1 h before sunset), and sunset (1 h before and after sunset). Sunrise and sunset times were based on the average times for sunrise and sunset in Xinjiang in 2023 (Appendix B: Table A2). Similarly, the JSI for each month was calculated.

The specaccum function from the Vegan package in R was used to generate the cumulative curve of camera working days and species numbers (Thompson and Withers 2003), effectively evaluating the relationship between monitoring time and species richness. The Kernel density estimation method was employed to construct and compare the utilization of wildlife passages across different time periods in sample areas, capturing the spatiotemporal distribution characteristics of wildlife activities (Tracey et al. 2014). The maximum retention method selected the highest RAI and SWI values from multiple monitoring points at each site to represent passage utilization.

Pearson correlation analysis was conducted on the influencing factors using SPSS 25.0 software. Variables with correlation coefficients |*r*| > 0.7 were excluded to mitigate the impact of multicollinearity on subsequent analyses (Schober, Boer, and Schwarte 2018). The results revealed significant correlations between habitat type and human activity intensity (*r*_*p*_ = 0.751, *p* < 0.01), as well as between habitat type and functional zone (*r*_*p*_ = 0.771, *p* < 0.01), and between traffic volume and linear infrastructure density (*r*_*p*_ = 0.789, *p* < 0.01). Consequently, habitat type and traffic volume were excluded from the analysis of wildlife passage utilization rates and their influencing factors. To ensure that multicollinearity did not affect the variables included in the redundancy analysis (RDA), the variance inflation factor (VIF) for the remaining variables was calculated, and variables with a VIF > 5 were excluded (Kutner et al. 2004). The inspection results indicated that all retained variables had VIF values below 5, specifically: passage size = 1.31, linear infrastructure density = 1.43, vegetation cover = 1.46, human activity intensity = 1.55, functional zone = 1.65, distance to water = 1.74, and passage type = 1.80. Ordination analysis was then conducted using Canoco 5 software. To select the appropriate ordination model, detrended correspondence analysis (DCA) was first performed to assess the relationship between species data and the gradient of influencing factors based on the length of the first DCA axis (Hill and Gauch 1980). Since the length of the first axis was < 3, indicating a linear gradient in the data, RDA was applied.

## 3 Results

### 3.1 Passage Construction Status

**FIGURE 3:**
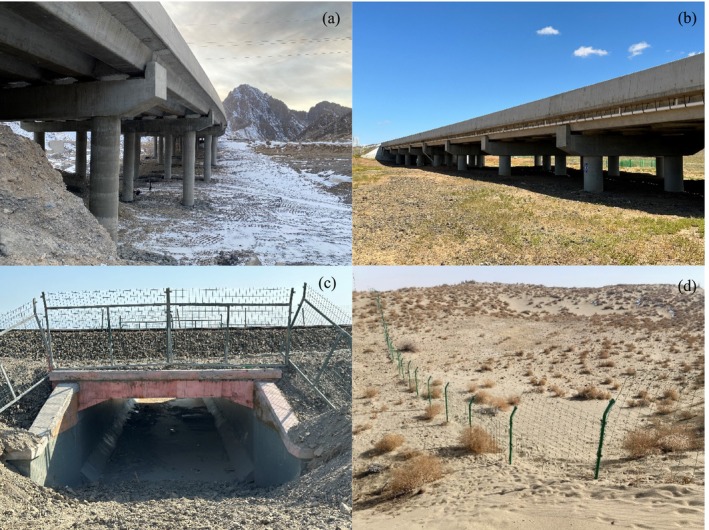
Typical wildlife passages and fences in nature reserves of Xinjiang. Panels (a–c) show wildlife passages located in the Lop Nur Wild Bactrian Camel National Nature Reserve, the Kalamaili Mountain Ungulate Wildlife National Nature Reserve, and the Tomur Peak National Nature Reserve, respectively. Panel (d) depicts a fence within the Peacock River Wetland Prefecture‐level Nature Reserve.

Large (0.1–1 million hm^2^) and extra‐large (> 1 million hm^2^) nature reserves, primarily designed to protect medium‐ and large‐sized mammals, especially ungulates, feature a number of dedicated wildlife passages, such as those found in the Lop Nur Wild Bactrian Camel Nature Reserve (Figure 3a) and the Kalamaili Mountain Ungulate Wildlife Nature Reserve (Figure 3b). These passages are characterized by larger clear widths, heights, and spans (Kal1, Kal2, Kal3, Kal4, Kal8, Lop1, Lop2, Lop3, and Lop4). Other nature reserves seldom have dedicated wildlife passages. Generally, considering geological and hydrological conditions, a number of small bridges and culverts were constructed to cross aquatic features, with some consideration of the passage needs of wild animals (Figure 3c). Road protective fences are typically installed in accordance with the “Design Specifications for Highway Traffic Safety Facilities” (JTG D81‐2017). However, due to the absence of clear construction standards for grassland fences (Pea, Sha), these fences typically only accommodate pedestrian passages and fail to consider the passage needs of wildlife. For example, the movement routes of wildlife, such as the goitered gazelle, Asiatic ibex, and argali, from Nanshan Mountain to Bosten Lake, are obstructed by the “maze” of fences constructed in the Peacock River Prefecture‐level Wetland Nature Reserve, Wetland Park, and Desertified Land Closed Reserve (Figure 3d).

### 3.2 Species Composition

**FIGURE 4:**
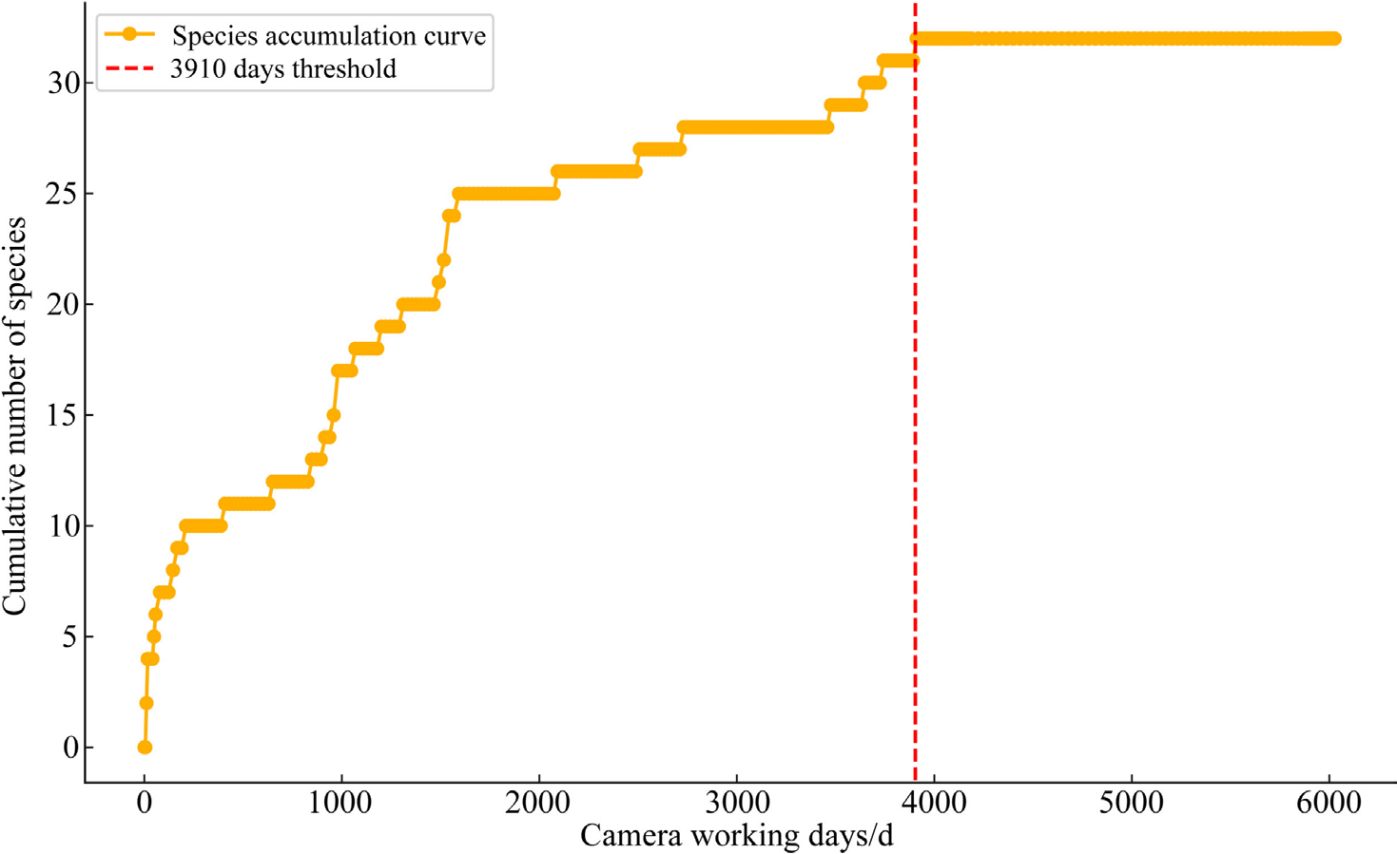
Cumulative curve of camera working days and species numbers.

**FIGURE 5:**
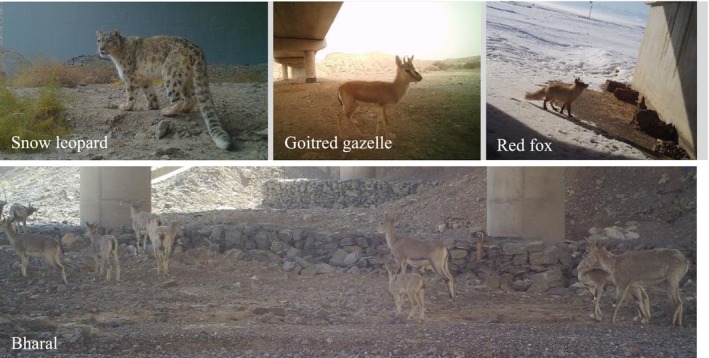
Some of the wildlife photos taken.

**FIGURE 6:**
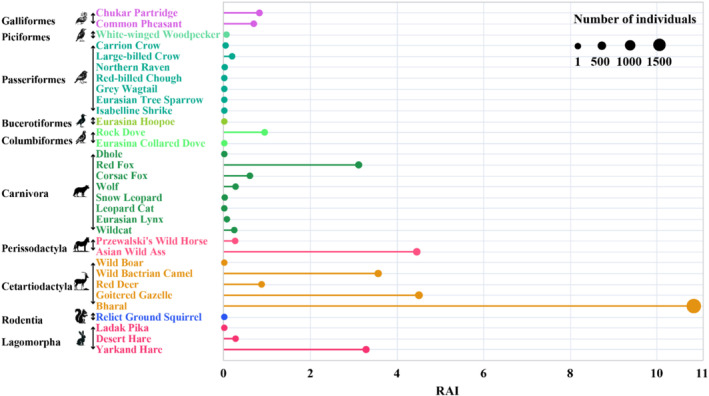
The RAI and the number of individuals observed. RAI, relative abundance index.

From February 2023 to January 2024, a total of 6027 camera working days resulted in 2143 independent valid wildlife photos. The analysis revealed that as monitoring days increased, the cumulative number of species gradually rose and stabilized after 3910 camera working days, with the number of newly recorded species significantly decreasing (difference ≤ 1). This suggests that the recorded species were sufficiently representative given the monitoring effort. Therefore, 3910 camera working days is recommended as the optimal duration for wildlife passage monitoring in Xinjiang (Figure 4). A total of 32 wildlife species were recorded (Table 3, Figure 5), including six first‐class and nine second‐class nationally protected species. Among these, 13 bird species were identified, spanning five orders and eight families, while 19 mammal species were recorded, representing 14.39% of Xinjiang's total mammal species and covering diverse orders such as Carnivora, Perissodactyla, and Cetancodonta. Species with relatively high RAI included the bharal, goitered gazelle, Asian wild ass, and wild Bactrian camel, with 2189, 358, 301, and 253 individuals detected, respectively (Figure 6). Additionally, the red fox and Yarkand hare were observed at multiple monitoring sites, with detection rates of 69.23% and 30.77%, respectively.

**TABLE 3:** Wildlife species list.

Order/Family/Species	Protection level	Monitoring sites/pcs (proportion/%)
I. Galliformes		
(1) Phasianidae		
1. Chukar Partridge ( *Alectoris chukar* )		12 (23.08)
2. Common Pheasant ( *Phasianus colchicus* )		2 (3.85)
II. Piciformes		
(2) Picidae		
3. White‐winged Woodpecker ( *Dendrocopos leucopterus* )	II	1 (1.92)
III. Passeriformes		
(3) Corvidae		
4. Carrion Crow ( *Corvus corone* )		1 (1.92)
5. Large‐billed Crow ( *Corvus macrorhynchos* )		2 (3.85)
6. Northern Raven ( *Corvus corax* )		1 (1.92)
7. Red‐billed Chough ( *Pyrrhocorax pyrrhocorax* )		1 (1.92)
(4) Motacillidae		
8. Gray Wagtail ( *Motacilla cinerea* )		1 (1.92)
(5) Passeridae		
9. Eurasian Tree Sparrow ( *Passer montanus* )		1 (1.92)
(6) Laniidae		
10. Isabelline Shrike ( *Lanius isabellinus* )		1 (1.92)
IV. Bucerotiformes		
(7) Upupidae		
11. Eurasian Hoopoe ( *Upupa epops* )		1 (1.92)
V. Columbiformes		
(8) Columbidae		
12. Rock Dove ( *Columba livia* )		4 (7.69)
13. Eurasian Collared Dove ( *Streptopelia decaocto* )		1 (1.92)
VI. Carnivora		
(9) Canidae		
14. Dhole ( *Cuon alpinus* )	I	1 (1.92)
15. Red Fox ( *Vulpes vulpes* )	II	36 (69.23)
16. Corsac Fox ( *Vulpes corsac* )	II	3 (5.77)
17. Wolf ( *Canis lupus* )	II	10 (19.23)
(10) Felidae		
18. Snow Leopard ( *Panthera uncia* )	I	2 (3.85)
19. Leopard Cat ( *Felis bengalensis* )	II	1 (1.92)
20. Eurasian Lynx ( *Lynx lynx* )	II	4 (7.69)
21. Wildcat ( *Felis silvestris* )		5 (9.62)
VII. Perissodactyla		
(11) Equidae		
22. Przewalski's Wild Horse ( *Equus ferus* )	I	3 (5.77)
23. Asian Wild Ass ( *Equus hemionus* )	I	14 (26.92)
VIII. Cetartiodactyla		
(12) Suidae		
24. Wild Boar ( *Sus scrofa* )		1 (1.92)
(13) Camelidae		
25. Wild Bactrian Camel ( *Camelus ferus* )	I	10 (19.23)
(14) Cervidae		
26. Red Deer (*Cervus elaphus Linnaeus*)	I	2 (3.85)
(15) Bovidae		
27. Goitered Gazelle ( *Gazella subgutturosa* )	II	24 (46.15)
28. Bharal ( *Pseudois nayaur* )	II	11 (21.15)
IX. Rodentia		
(16) Sciuridae		
29. Relict Ground Squirrel ( *Spermophilus relictus* )		1 (1.92)
X. Lagomorpha		
(17) Ochotonidae		
30. Ladak Pika ( *Ochotona ladacensis* )		1 (1.92)
(18) Leporidae		
31. Desert Hare ( *Lepus tibetanus* )		6 (11.54)
32. Yarkand Hare ( *Lepus yarkandensis* )	II	16 (30.77)

### 3.3 Wildlife Passage Utilization Patterns

**FIGURE 7:**
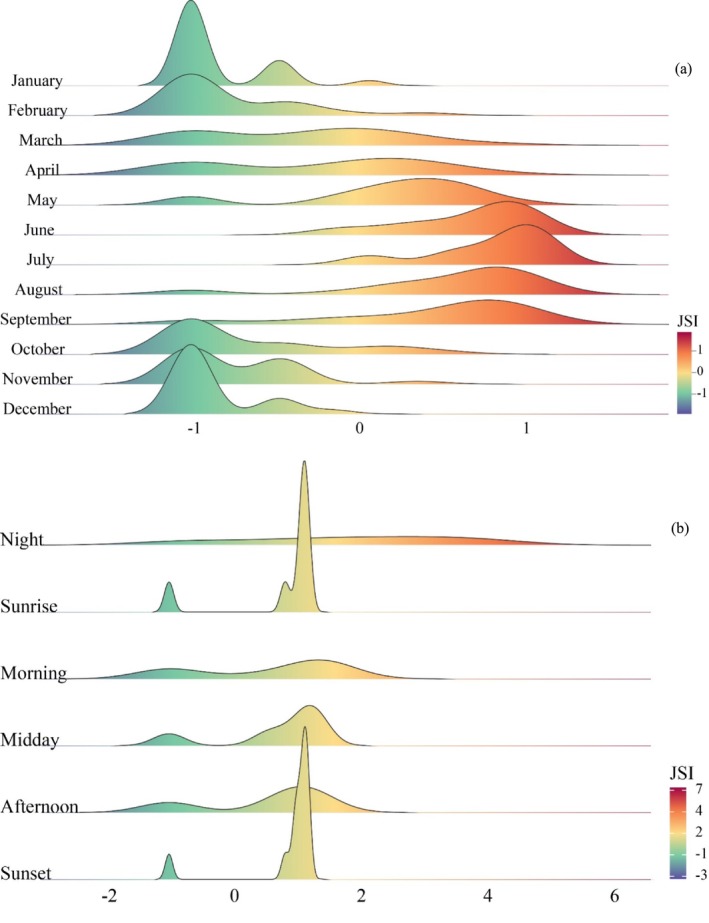
The JSI at different periods. JSI, Jacobs selection index. Panels (a) show the JSI index for each month. Panels (b) showthe JSI index for different time periods of the day.

Wildlife passage utilization exhibited notable seasonal variation (Table 4, Figure 7a). The utilization rate was lowest in winter, followed by a marked increase in spring, and sustained high levels during the summer and autumn. In July, all monitoring sites showed active selection (JSI > 0), while 69.57%, 91.3%, 86.96%, and 69.57% of the monitoring sites exhibited active selection in May, June, August, and September, respectively. Additionally, notable regional differences in passage utilization were observed between southern and northern Xinjiang. In southern Xinjiang, the Lop Nur Wild Bactrian Camel National Nature Reserve exhibited the highest passage utilization, with JSI values of 1.1, 1.17, and 1.12 for passages Lop3, Lop4, and Lop5 in July, respectively. In northern Xinjiang, the Karakol Mountain Ungulate Wildlife National Nature Reserve exhibited the second‐highest utilization, with the Kal2 passage recording a JSI of 1.08 in July. Bird passage utilization was especially prominent during the migration season, peaking in April when 57.58% of the total bird individuals recorded throughout the year were observed. This was followed by March (13.42%) and September (12.99%). In the Bayingbrook National Nature Reserve, which focuses on the conservation of rare waterfowl and their breeding habitats, bird passage utilization was highest during the migration season.

**TABLE 4:** JSI for various months at monitoring sites.

Site code	January	February	March	April	May	June	July	August	September	October	November	December
Bay1	−1.00	−1.00	0.07	0.09	0.35	0.83	0.96	0.77	0.68	−0.15	−0.46	−0.47
Bay2	−1.00	−1.00	−1.00	−1.00	−1.00	−0.13	0.07	−1.00	−1.00	−1.00	−1.00	−1.00
Kal1	−0.47	−0.43	−0.15	0.09	0.44	0.93	1.03	0.87	0.83	0.07	−1.00	−1.00
Kal2	−0.47	−0.09	0.23	0.25	0.58	0.99	1.08	0.96	0.91	−0.47	−0.46	−1.00
Kal3	−0.47	−1.00	−0.15	−0.13	0.23	0.78	0.93	0.71	0.64	−1.00	−1.00	−1.00
Kal4	−0.47	−1.00	−0.15	−0.13	0.23	0.80	0.94	0.74	0.64	−1.00	−1.00	−1.00
Kal5	−1.00	−1.00	−1.00	−1.00	−1.00	−0.13	0.07	−1.00	−1.00	−1.00	−1.00	−1.00
Kal6	−1.00	−1.00	−1.00	−1.00	0.07	0.36	0.58	0.35	−0.13	−1.00	−1.00	−1.00
Kal7	−1.00	−1.00	−1.00	−1.00	−0.15	0.36	0.58	0.23	−0.13	−1.00	−1.00	−1.00
Kal8	−1.00	−1.00	−1.00	−1.00	−1.00	0.09	0.07	−0.15	−0.46	−1.00	−0.46	−0.47
Pea1	−1.00	−0.43	0.23	0.36	0.63	0.99	1.08	0.96	0.91	−0.47	−0.46	−1.00
Lop1	−1.00	−1.00	−0.15	0.09	0.44	0.85	0.98	0.77	0.72	−1.00	−1.00	−1.00
Lop2	−1.00	−1.00	−0.15	0.25	0.52	0.93	1.03	0.87	0.83	−0.47	−1.00	−1.00
Lop3	−1.00	−0.43	0.23	0.46	0.71	1.02	1.10	0.99	0.95	0.23	−0.46	−1.00
Lop4	0.07	0.40	0.84	0.83	1.00	1.14	1.17	1.13	1.11	0.52	0.36	−0.15
Lop5	−0.47	−0.43	0.44	0.53	0.77	1.05	1.12	1.03	0.99	0.23	−0.46	−1.00
Lop6	−1.00	−1.00	−1.00	−0.13	0.07	0.59	0.74	0.44	0.46	−1.00	−0.46	−0.47
Lop7	−1.00	−1.00	−0.15	0.25	0.44	0.86	0.99	0.77	0.75	−1.00	−0.46	−0.47
Lop8	−1.00	−1.00	−0.47	−1.00	0.23	0.64	0.82	0.58	0.25	−1.00	−1.00	−1.00
Nil	−1.00	−1.00	−1.00	−1.00	−0.15	0.25	0.52	0.23	0.09	−1.00	−1.00	−1.00
Sha	−1.00	−1.00	−0.47	−0.46	0.35	0.80	0.95	0.74	0.68	−1.00	−1.00	−1.00
Tar	−1.00	−0.43	0.23	0.36	0.67	0.99	1.08	0.96	0.92	−0.47	−0.46	−1.00
Tom	−1.00	−1.00	−1.00	−1.00	0.07	0.46	0.63	0.23	0.36	−1.00	−1.00	−1.00

Nighttime passage utilization followed an unimodal distribution, significantly exceeding other periods (Table 5, Figure 7b). At night, wildlife exhibited the strongest preference for the overpass at the Pea passage in the Peacock River Wetland Prefecture‐level Nature Reserve (JSInight = 4.19) and the underpass at the Kal2 passage in the Karakol Mountain Ungulate Wildlife National Nature Reserve (JSInight = 4.08). In contrast, passage utilization during other periods exhibited a bimodal distribution, with distinct peaks at sunrise and sunset. At sunrise, 86.96% of the monitoring sites showed JSI > 0, while at sunset, 91.3% of the monitoring sites showed JSI > 0. During the daytime, no significant differences in wildlife passage utilization were observed at the Lop Nur Wild Bactrian Camel National Nature Reserve and the Tarim Populus Euphratica National Nature Reserve. For birds, the highest passage utilization occurred at noon (45.02%), followed by the afternoon (25.11%) and morning (21.64%).

**TABLE 5:** JSI for different time periods at monitoring sites.

Site code	Night	Sunrise	Morning	Midday	Afternoon	Sunset
Bay1	0.20	1.12	1.42	1.30	1.02	1.12
Bay2	−0.46	−1.00	−1.00	0.56	−1.00	−1.00
Kal1	3.86	1.17	0.55	−1.00	0.64	1.18
Kal2	4.08	1.18	1.27	1.15	1.16	1.18
Kal3	2.40	1.14	1.19	1.18	0.91	1.16
Kal4	0.89	1.14	1.42	1.24	1.22	1.06
Kal5	−0.72	0.83	−1.00	−1.00	−1.00	0.83
Kal6	1.30	0.83	−1.00	0.56	−1.00	1.12
Kal7	1.17	−1.00	−1.00	−1.00	0.91	1.10
Kal8	−1.00	0.83	0.10	0.87	0.91	1.00
Pea1	4.19	1.16	1.30	1.15	1.16	1.18
Lop1	3.59	1.16	0.55	0.56	−1.00	1.13
Lop2	2.59	1.12	1.46	1.30	1.12	1.00
Lop3	2.65	1.15	1.56	1.26	1.26	1.13
Lop4	2.47	1.12	1.60	1.33	1.27	1.18
Lop5	3.21	1.18	1.56	1.30	1.26	1.17
Lop6	−1.00	1.06	1.30	1.22	1.18	1.00
Lop7	1.74	1.10	1.43	1.26	1.22	1.16
Lop8	0.39	1.10	1.32	1.23	1.02	1.00
Nil	1.53	−1.00	−1.00	−1.00	−1.00	0.83
Sha	3.54	1.15	−1.00	0.87	0.64	−1.00
Tar	3.42	1.18	1.44	1.29	1.24	1.17
Tom	1.93	1.06	−1.00	0.56	−1.00	1.00

### 3.4 Identification of Major Influencing Factors

**FIGURE 8:**
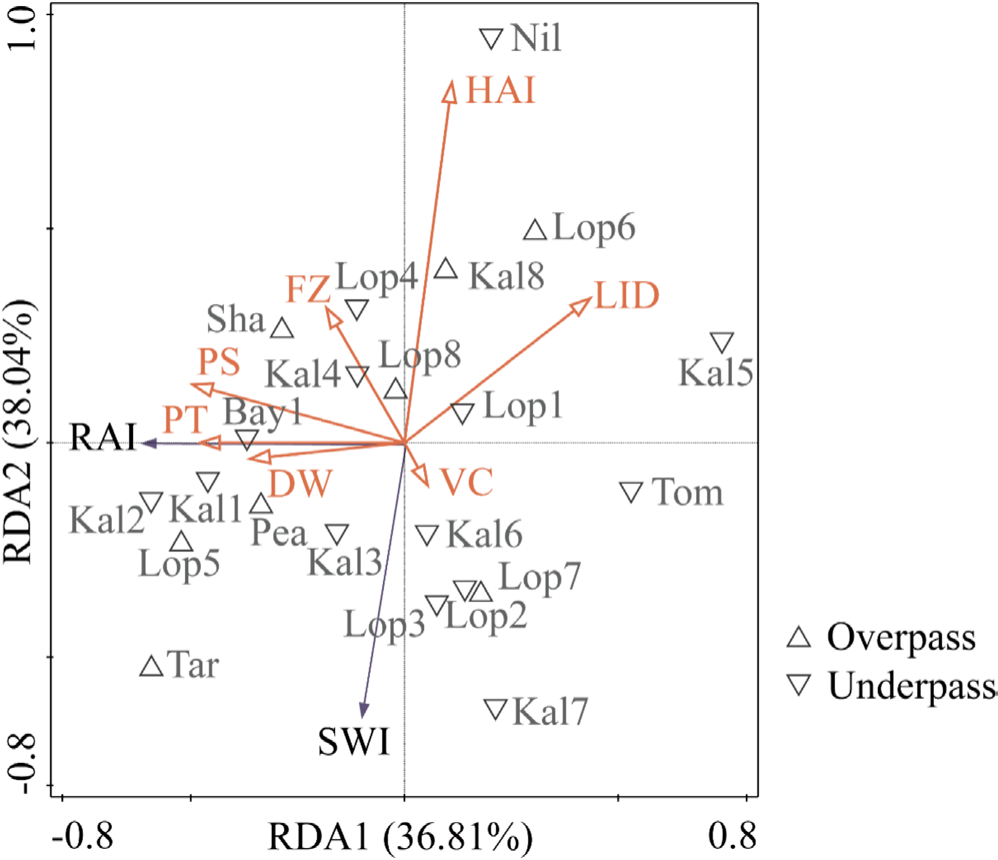
RDA analysis of wildlife passage utilization rates and influencing factors. DW, Distance from water; FZ, functional zone; HAI, human activity intensity; LID, linear infrastructure density; PS, passage size; PT, passage type; RAI, relative abundance index; SWI, Shannon–Wiener index; VC, vegetation coverage.

As shown in Figure 8, the results of the RDA analysis for all monitoring sites indicate that the first two axes, RDA1 and RDA2, explain 36.81% and 38.04% of the variation, respectively, facilitating the construction of an effective two‐dimensional ordination plot. The analysis revealed that human activity intensity and linear infrastructure density were negatively correlated with both the RAI and SWI, suggesting that lower levels of human activity and linear infrastructure density are associated with higher wildlife passage utilization. In contrast, passage size, passage type, and distance from water were positively correlated with the RAI, indicating that larger passages and those closer to water sources are associated with higher utilization rates. Additionally, the utilization of overpasses (RAI = 72.83) was significantly higher than that of underpasses (RAI = 43.36), highlighting the impact of passage structure on its utilization efficiency. Vegetation cover was also positively correlated with the SWI, implying that areas with higher vegetation cover support greater species diversity within the passages.

Regarding passage types, underpasses, often consisting of elevated bridges, tend to be larger than overpasses, which are typically composed of fences. Furthermore, the surrounding linear infrastructure density and human activity intensity around underpasses are generally higher than those around overpasses, leading to lower RAI and SWI values for underpasses. In terms of functional zoning, increasing protection intensity tends to be associated with greater distances between wildlife passages and water sources, which correlates with a decrease in SWI. In contrast, passages located within core protection zones exhibited higher RAI values. In conclusion, wildlife passages in the Tarim Populus Euphratica National Nature Reserve, located within the core protection zone and consisting of overpasses, exhibited the highest RAI and SWI. This area had low linear infrastructure density and human activity intensity, as well as the highest surrounding vegetation cover (34.2%). In comparison, the Dense‐Leaved Poplar, County‐Level Nature Reserve in the Nilekkashgar River Basin, with significantly higher human activity intensity, had the lowest SWI and relatively low RAI.

## 4 Discussion

This study investigated nature reserves that encompass a diverse range of ecosystems, including deserts, grasslands, forests, and wetlands. Using a combination of stratified and systematic sampling methods to establish monitoring points, the study ensured coverage across a variety of habitats and areas of high wildlife activity, thus yielding comprehensive and representative data. In collaboration with management agencies, the layout of monitoring points was optimized, and infrared camera technology was employed to obtain reliable and precise monitoring data (Cilulko et al. 2013; Swann et al. 2011). The findings indicated that larger nature reserves were equipped with more dedicated wildlife passages, effectively facilitating the migration and movement of medium‐ and large‐sized mammals, such as ungulates. In contrast, smaller reserves primarily depended on culverts and water‐crossing bridges, which were insufficient to fully accommodate the migratory needs of wildlife. This highlights the urgent need to expand and enhance passage infrastructure, particularly along critical migratory routes of protected species. The study documented 32 wildlife species, including 15 species listed as nationally protected, emphasizing the region's rich biodiversity. These findings not only validate the effectiveness of existing wildlife passages but also provide a scientific foundation for optimizing and constructing additional passage infrastructure to further enhance biodiversity conservation.

The type of wildlife passage plays a pivotal role in determining species composition and usage patterns. Underpasses, typically designed as elevated bridges, are large, open, and spacious, making them particularly suitable for medium‐ and large‐sized mammals. Monitoring data indicate that bharal, Asian wild ass, and wild Bactrian camel are the primary users of underpasses, with the highest RAI values recorded at these sites. These structures provide ample space and unobstructed sightlines, reducing psychological barriers and significantly enhancing the safety of migration and foraging (Clevenger and Waltho 2005; Donaldson 2007). In contrast, overpasses, often simple structures with fenced reservations, are smaller in scale and more suitable for small mammals and ground‐dwelling birds, such as the chukar partridge. However, the lack of vegetation cover and concealment limits their functionality to facilitating short‐distance movements rather than supporting long‐range migrations. Culvert passages, on the other hand, are predominantly utilized by rodents, lagomorphs, and small carnivores such as the red fox and corsac fox. Although culverts are smaller in size with limited light and airflow, their concealed nature is critical for ensuring the safe passage of smaller species (Martinig and Bélanger‐Smith 2016). Each type of passage offers unique advantages in supporting species diversity and migratory behaviors, collectively contributing to a multilayered ecological functional network. Future passage constructions should be guided by the ecological needs and behavioral characteristics of regional species, with the spatial distribution of different passage types optimized to achieve biodiversity conservation objectives more effectively.

The utilization of wildlife passages is influenced by seasonal variations, time of day, and human activities (Xi et al. 2020; Whittington, Hebblewhite, and Baron 2022). This study observed significantly higher passage usage in summer and autumn compared to spring and winter, corresponding to increased wildlife activity during seasons with abundant food resources. Passage usage peaked at night, with a strong preference for dawn and dusk, suggesting an avoidance of daytime human disturbances. During the daytime, increased disturbances from motor vehicle traffic, road maintenance, patrols, grazing, and tourism led to passages being predominantly utilized by widely distributed species such as the goitered gazelle, Asian wild ass, bharal, and Yarkand hare. In contrast, in areas with lower human activity intensity, such as the Lop Nur Wild Bactrian Camel National Nature Reserve and the core zones of the Tarim Populus Euphratica National Nature Reserve, temporal differences in passage use were less evident. In conclusion, the construction and management of wildlife passages should carefully consider seasonal and temporal patterns, prioritizing the reduction of human disturbance at night and during dawn and dusk. This approach is crucial for improving passage utilization and ensuring that the migration and survival needs of wildlife are adequately met.

The density and technical specifications of linear infrastructure significantly affect the efficiency of wildlife passage utilization. This study found that passages in areas with low‐density linear infrastructure exhibited higher utilization rates, supporting the adverse effects of habitat fragmentation on wildlife behavior (Fahrig 2003; Li et al. 2022). Additionally, high‐grade roads, such as highways, characterized by high traffic volumes and substantial physical barriers, were particularly detrimental to passage utilization and habitat connectivity. To address these challenges, wildlife passage construction should be tailored to the density and technical grade of linear infrastructure. For high‐grade roads, passage construction should prioritize factors such as width, spacing, and the number of passages to accommodate the behavioral needs of various species. Optimization strategies may include increasing the number of passages, scientifically adjusting their spacing, and incorporating fence installations to mitigate the disruptive effects of infrastructure on wildlife movement. Fences, in particular, significantly affect wildlife movement, especially in areas with low passage density, where their barrier effect is more pronounced. To mitigate this, passage density in fenced areas should be increased, and habitat connectivity improved by adding more passages. Implementing dynamic fence management, such as seasonally opening passages based on migration periods and ecological needs, could further improve passage efficiency. Furthermore, effective coordination of wildlife passages both within and beyond nature reserves requires the establishment of regional passage management policies. Within the core and buffer zones of nature reserves, efforts should focus on strengthening the protection and maintenance of passages to preserve the integrity of critical habitats. Simultaneously, promoting the construction of ecological corridors outside nature reserves can mitigate the negative impacts of linear infrastructure in crossing zones. This dual approach would not only provide safer migration routes and support population connectivity for wildlife but also enhance the stability and connectivity of regional ecosystems.

Xinjiang's diverse topography and pronounced climatic variations profoundly shape wildlife passage utilization and behavioral patterns. In southern Xinjiang, the arid climate, marked by extremely low annual precipitation and high summer temperatures, renders water sources a critical limiting factor. This compels species such as the goitered gazelle, red deer, bharal, and wild Bactrian camel to rely more heavily on passages during dry seasons to access water and sustain survival. In contrast, northern Xinjiang experiences a temperate continental arid to semi‐arid climate with distinct seasons and comparatively higher rainfall. Seasonal vegetation changes in this region offer diverse ecological resources, encouraging migratory species such as Przewalski's wild horse and Asian wild ass to utilize passages more frequently. Future research should integrate climatic variables—including precipitation, temperature, and evapotranspiration—with wildlife passage utilization data to uncover the dynamic interplay between climatic conditions and wildlife behavior across Xinjiang's varied regions. This approach would yield deeper insights into the spatiotemporal patterns of passage use and guide more effective conservation strategies.

Current multispecies studies exhibit notable limitations, particularly in addressing species such as amphibians and reptiles. These groups exhibit high sensitivity to passage microenvironments, including factors such as humidity, temperature, and structural dimensions. However, due to limitations in monitoring technologies and the selective focus on target species, the passage utilization patterns of amphibians and reptiles remain insufficiently explored in many studies (Testud et al. 2020). This study primarily examined the passage use patterns of birds and mammals in Xinjiang, addressing specific regional gaps in multispecies research. However, it did not encompass all animal groups, particularly amphibians and reptiles, thus underscoring the need for more comprehensive monitoring in future studies. Additionally, this study focused on terrestrial wildlife passages, with limited consideration of aquatic migration corridors. Many nature reserves include critical aquatic habitats, where the construction and operation of water infrastructure can have significant impacts on migratory species. Future research should prioritize nature reserves with significant aquatic migration corridors to investigate the effects of water infrastructure on migratory species and assess the effectiveness of conservation measures. Such research will contribute to addressing these overlooked challenges in a comprehensive manner, ensuring the conservation of aquatic species diversity and the overall health of ecosystems.

## 5 Conclusions

This study systematically identifies the utilization patterns of wildlife passages in Xinjiang, along with the key factors influencing their use. The study found that passage utilization rates are significantly affected by human activity intensity and linear infrastructure density, while factors such as passage type, size, and proximity to water also play crucial roles. The high‐frequency use of passages by birds during migration and by mammals in summer and autumn reflects species' dynamic adaptation to resources and habitats. Based on these findings, it is recommended to optimize the construction and spatial arrangement of passages, particularly through the implementation of dynamic management in fenced grassland areas to enhance passage density and functionality. Future research should prioritize the passage requirements of amphibians, reptiles, and aquatic species. Furthermore, collaborative management policies both within and beyond nature reserves should be developed. Long‐term monitoring and technological innovations will be essential in providing scientific evidence and practical guidance for the coordinated development of wildlife conservation and infrastructure in Xinjiang.

## Author Contributions

**Mengdi Fu:** data curation (lead), formal analysis (lead), funding acquisition (lead), investigation (lead), visualization (lead), writing – original draft (lead). **Jun Wang:** data curation (supporting), methodology (equal), software (lead). **Shuang Li:** formal analysis (supporting), investigation (equal). **Le Qin:** investigation (equal), visualization (supporting). **Junsheng Li:** methodology (equal), writing – review and editing (equal). **Shichao Jin:** resources (lead), writing – review and editing (lead).

## Ethics Statement

Field investigations were conducted in compliance with ethical standards and were approved by the Nature Reserve Authority.

## Conflicts of Interest

The authors declare no conflicts of interest.

## Data Availability

The data that support the findings of this study are available from the Dryad (https://doi.org/10.5061/dryad.ncjsxkt4v).

## Appendix A

**TABLE A1:** The performance and settings of the cameras.

Infrared camera performance and settings		
Performance	Parameter	Description
Data quality	Shooting mode	Three photos
	Physical pixels	12 million
Start‐up speed	Trigger to shooting time	0.8 s
	The minimum interval between continuous shots of the image	1 s
	Trigger sensitivity.	Middle
Power consumption	Standby time	6 ~ 8 months
	Operating temperature	−30°C ~ 70°C Can operate normally
Hardware	Fill light system	At night and in low‐light conditions, the fill light system is automatically activated to assist in shooting
	Protective performance	IP68^a^

^a^ IP68 is the highest level of dust and water resistance standard in GB/T 4208–2017 “Enclosure Protection Rating (IP Code)”.

## Appendix B

**TABLE A2:** Sunrise and Sunset Times in Xinjiang in 2023.

Statistics of sunrise and sunset times in Xinjiang in 2023		
Month	Sunrise time	Sunset time
January	09:35	19:00
February	09:07	19:37
March	08:19	20:15
April	07:27	20:52
May	06:45	21:27
June	06:30	21:49
July	06:43	21:44
August	07:14	21:10
September	07:48	20:19
October	08:24	19:26
November	09:03	18:47
December	09:33	18:37

## References

[ece370969-bib-0001] Baechli, J. , S. Albanesi , and L. M. Bellis . 2021. “Effectiveness of Crossings as Wildlife Passages for Mammals in the Yungas of Argentina.” Journal for Nature Conservation 59: 125944.

[ece370969-bib-0002] Bakaloudis, D. E. , V. A. Bontzorlos , and E. Kotsonas . 2023. “Wildlife Mortality on Roads Crossing a Protected Area: The Case of Dadia‐Lefkimi‐Soufli National Park in North‐Eastern Greece.” Journal for Nature Conservation 74: 126443.

[ece370969-bib-0003] Barrueto, M. , A. T. Ford , and A. P. Clevenger . 2014. “Anthropogenic Effects on Activity Patterns of Wildlife at Crossing Structures.” Ecosphere 5: 1–19.

[ece370969-bib-0004] Campbell, M. D. , A. G. Pollack , C. T. Gledhill , T. S. Switzer , and D. A. DeVries . 2015. “Comparison of Relative Abundance Indices Calculated From Two Methods of Generating Video Count Data.” Fisheries Research 170: 125–133.

[ece370969-bib-0005] Cilulko, J. , P. Janiszewski , M. Bogdaszewski , and E. Szczygielska . 2013. “Infrared Thermal Imaging in Studies of Wild Animals.” European Journal of Wildlife Research 59: 17–23.

[ece370969-bib-0006] Clevenger, A. P. , B. Chruszcz , and K. Gunson . 2001. “Drainage Culverts as Habitat Linkages and Factors Affecting Passage by Mammals.” Journal of Applied Ecology 38: 1340–1349.

[ece370969-bib-0007] Clevenger, A. P. , and N. Waltho . 2000. “Factors Influencing the Effectiveness of Wildlife Underpasses in Banff National Park, Alberta, Canada.” Conservation Biology 14: 47–56.

[ece370969-bib-0008] Clevenger, A. P. , and N. Waltho . 2005. “Performance Indices to Identify Attributes of Highway Crossing Structures Facilitating Movement of Large Mammals.” Biological Conservation 121: 453–464.

[ece370969-bib-0009] Coffin, A. W. 2007. “From Roadkill to Road Ecology: A Review of the Ecological Effects of Roads.” Journal of Transport Geography 15: 396–406.

[ece370969-bib-0010] Collinson, W. J. , C. Marneweck , and H. T. Davies‐Mostert . 2019. “Protecting the Protected: Reducing Wildlife Roadkill in Protected Areas.” Animal Conservation 22: 396–403.

[ece370969-bib-0011] Dodd, C. K., Jr. , W. J. Barichivich , and L. L. Smith . 2004. “Effectiveness of a Barrier Wall and Culverts in Reducing Wildlife Mortality on a Heavily Traveled Highway in Florida.” Biological Conservation 118, no. 5: 619–631. 10.1016/j.biocon.2003.10.011.

[ece370969-bib-0012] Donaldson, B. 2007. “Use of Highway Underpasses by Large Mammals and Other Wildlife in Virginia: Factors Influencing Their Effectiveness.” Transportation Research Record 2011: 157–164.

[ece370969-bib-0013] Fahrig, L. 2003. “Effects of Habitat Fragmentation on Biodiversity.” Annual Review of Ecology, Evolution, and Systematics 34: 487–515.

[ece370969-bib-0014] Forman, R. T. T. , and L. E. Alexander . 1998. “Roads and Their Major Ecological Effects.” Annual Review of Ecology and Systematics 29: 207–231.

[ece370969-bib-0015] Gagnon, J. W. , N. L. Dodd , K. S. Ogren , and R. E. Schweinsburg . 2011. “Factors Associated With Use of Wildlife Underpasses and Importance of Long‐Term Monitoring.” Journal of Wildlife Management 75: 1477–1487.

[ece370969-bib-0016] Geneletti, D. 2004. “Using Spatial Indicators and Value Functions to Assess Ecosystem Fragmentation Caused by Linear Infrastructures.” International Journal of Applied Earth Observation and Geoinformation 5: 1–15.

[ece370969-bib-0017] Glista, D. J. , T. L. DeVault , and J. A. DeWoody . 2009. “A Review of Mitigation Measures for Reducing Wildlife Mortality on Roadways.” Landscape and Urban Planning 91: 1–7.

[ece370969-bib-0018] Gloyne, C. C. , and A. P. Clevenger . 2001. “Cougar *Puma Concolor* Use of Wildlife Crossing Structures on the Trans‐Canada Highway in Banff National Park, Alberta.” Wildlife Biology 7: 117–124.

[ece370969-bib-0019] Gunson, K. E. , G. Mountrakis , and L. J. Quackenbush . 2011. “Spatial Wildlife‐Vehicle Collision Models: A Review of Current Work and Its Application to Transportation Mitigation Projects.” Journal of Environmental Management 92: 1074–1082. 21190788 10.1016/j.jenvman.2010.11.027

[ece370969-bib-0020] Hill, M. O. , and H. G. Gauch . 1980. “Detrended Correspondence Analysis: An Improved Ordination Technique.” Vegetatio 42: 47–58.

[ece370969-bib-0021] Hull, V. , W. Xu , W. Liu , et al. 2011. “Evaluating the Efficacy of Zoning Designations for Protected Area Management.” Biological Conservation 144: 3028–3037.

[ece370969-bib-0022] Jackson, S. D. , and C. R. Griffin . 2000. “A Strategy for Mitigating Highway Impacts on Wildlife.” In Wildlife and Highways: Seeking Solutions to an Ecological and Socio‐Economic Dilemma, edited by T. A. Messmer and B. West , 143–159. Wildlife Society.

[ece370969-bib-0023] Jacobs, J. 1974. “Quantitative Measurement of Food Selection: A Modification of the Forage Ratio and Ivlev's Electivity Index.” Oecologia 14: 413–417. 28308662 10.1007/BF00384581

[ece370969-bib-0024] Jacobson, S. L. , L. L. Bliss‐Ketchum , C. E. de Rivera , and W. P. Smith . 2016. “A Behavior‐Based Framework for Assessing Barrier Effects to Wildlife From Vehicle Traffic Volume.” Ecosphere 7: e01345.

[ece370969-bib-0025] Kühl, H. S. , S. T. Buckland , M. Henrich , E. Howe , and M. Heurich . 2023. “Estimating Effective Survey Duration In Camera Trap Distance Sampling Surveys.” Ecology and Evolution 13: e10599. 37841220 10.1002/ece3.10599 PMC10571013

[ece370969-bib-0026] Kutner, M. H. , C. J. Nachtsheim , J. Neter , and W. Wasserman . 2004. Applied Linear Regression Models. McGraw‐Hill.

[ece370969-bib-0027] Li, G. , C. Fang , Y. Li , et al. 2022. “Global Impacts of Future Urban Expansion on Terrestrial Vertebrate Diversity.” Nature Communications 13: 1628. 10.1038/s41467-022-29324-2 PMC8956596 35338145

[ece370969-bib-0028] Malo, J. E. , I. Hervás , J. Herranz , C. Mata , and F. Suárez . 2005. “How Many Days to Monitor a Wildlife Passage? Species Detection Patterns and the Estimation of the Vertebrate Fauna Using Crossing Structures at a Motorway.” In Proceedings of the 2005 International Conference on Ecology and Transportation, edited by C. L. Irwin , D. Nelson , and K. P. McDermott , 406–413. Center for Transportation and the Environment, North Carolina State University.

[ece370969-bib-0029] Martinig, A. R. , and K. Bélanger‐Smith . 2016. “Factors Influencing the Discovery and Use of Wildlife Passages for Small Fauna.” Journal of Applied Ecology 53: 825–836.

[ece370969-bib-0030] Mata, C. , I. Hervás , J. Herranz , F. Sua'rez , and J. E. Malo . 2008. “Are Motorway Wildlife Passages Worth Building? Vertebrate Use of Road‐Crossing Structures on a Spanish Motorway.” Journal of Environmental Management 88: 407–415. 17467145 10.1016/j.jenvman.2007.03.014

[ece370969-bib-0031] McCollister, M. F. , and F. T. van Manen . 2010. “Effectiveness of Wildlife Underpasses and Fencing to Reduce Wildlife‐Vehicle Collisions.” Journal of Wildlife Management 74: 1722–1731.

[ece370969-bib-0032] Mulualem, G. , W. J. C. Jonker , W. Tesfahunegny , and M. Wale . 2023. “Examining Vertebrate Road Mortality on Highways Passing Through Protected Areas of Eastern Ethiopia.” European Journal of Wildlife Research 69: 117.

[ece370969-bib-0033] Mysłajek, R. W. , E. Olkowska , M. Wronka‐Tomulewicz , and S. Nowak . 2020. “Mammal Use of Wildlife Crossing Structures Along a New Motorway in an Area Recently Recolonized by Wolves.” European Journal of Wildlife Research 66: 79.

[ece370969-bib-0034] Ng, S. J. , J. W. Dole , R. M. Sauvajot , S. P. D. Riley , and T. J. Valone . 2004. “Use of Highway Undercrossings by Wildlife in Southern California.” Biological Conservation 115: 499–507.

[ece370969-bib-0035] Ramita, M. , I. O. A. Odeh , and A. Tiho . 2009. “Improving the Accuracy of Land Use and Land Cover Classification of Landsat Data Using Post‐Classification Enhancement.” Remote Sensing 1: 330–344.

[ece370969-bib-0036] Ree, R. , J. A. G. Jaeger , E. A. Grift , and A. P. Clevenger . 2011. “Effects of Roads and Traffic on Wildlife Populations and Landscape Function: Road Ecology Is Moving Toward Larger Scales.” Ecology and Society 16: 48.

[ece370969-bib-0037] Rytwinski, T. , R. van der Ree , G. M. Cunnington , et al. 2015. “Experimental Study Designs to Improve the Evaluation of Road Mitigation Measures for Wildlife.” Journal of Environmental Management 154: 48–64. 10.1016/j.jenvman.2015.01.048. 25704749

[ece370969-bib-0038] Schober, P. , C. Boer , and L. A. Schwarte . 2018. “Correlation Coefficients: Appropriate Use and Interpretation.” Anesthesia & Analgesia 126: 1763–1768. 29481436 10.1213/ANE.0000000000002864

[ece370969-bib-0039] Seidler, R. G. , D. S. Green , and J. P. Beckmann . 2018. “Highways, Crossing Structures and Risk: Behaviors of Greater Yellowstone Pronghorn Elucidate Efficacy of Road Mitigation.” Global Ecology and Conservation 15: e00416.

[ece370969-bib-0040] Sluijs, J. V. D. , G. Mackay , L. Andrew , N. Smethurst , and T. D. Andrews . 2020. “Archaeological Documentation of Wood Caribou Fences Using Unmanned Aerial Vehicle and Very High‐Resolution Satellite Imagery in the Mackenzie Mountains, Northwest Territories.” Journal of Unmanned Vehicle Systems 8, no. 3: 186–206. 10.1139/juvs-2020-0007.

[ece370969-bib-0041] Strong, W. L. 2016. “Biased Richness and Evenness Relationships Within Shannon‐Wiener Index Values.” Ecological Indicators 67: 703–713.

[ece370969-bib-0042] Swann, D. E. , C. C. Hass , D. C. Dalton , and S. A. Wolf . 2011. “Infrared‐Triggered Cameras for Detecting Wildlife: An Evaluation and Review.” Wildlife Society Bulletin 32: 357–365.

[ece370969-bib-0043] Testud, G. , C. Fauconnier , D. Labarraque , et al. 2020. “Acoustic Enrichment in Wildlife Passages Under Railways Improves Their Use by Amphibians.” Global Ecology and Conservation 24: e01252.

[ece370969-bib-0044] Thompson, G. G. , and P. C. Withers . 2003. “Effect of Species Richness and Relative Abundance on the Shape of the Species Accumulation Curve.” Austral Ecology 28: 355–360.

[ece370969-bib-0045] Tong, C. 2006. “Refinement Strategies for Stratified Sampling Methods.” Reliability Engineering and System Safety 91: 1257–1265.

[ece370969-bib-0046] Tracey, J. A. , J. Sheppard , J. Zhu , F. Wei , R. R. Swaisgood , and R. N. Fisher . 2014. “Movement‐Based Estimation and Visualization of Space Use in 3D for Wildlife Ecology and Conservation.” PLoS One 9: e109065. 10.1371/journal.pone.0101205 PMC4079284 24988114

[ece370969-bib-0047] Trombulak, S. C. , and C. A. Frissell . 2000. “Review of Ecological Effects of Roads on Terrestrial and Aquatic Communities.” Conservation Biology 14: 18–30.

[ece370969-bib-0048] Underhill, J. E. , and P. G. Angold . 1999. “Effects of Roads on Wildlife in an Intensively Modified Landscape.” Environmental Reviews 8: 21–39.

[ece370969-bib-0049] Wang, Y. , L. Guan , J. Chen , and Y. Kong . 2018. “Influences on Mammals Frequency of Use of Small Bridges and Culverts Along the Qinghai‐Tibet Railway, China.” Ecological Research 33: 879–887.

[ece370969-bib-0050] Wang, Y. , L. Guan , Z. Piao , Z. Wang , and Y. Kong . 2017. “Monitoring Wildlife Crossing Structures Along Highways in Changbai Mountain, China.” Transportation Research Part D: Transport and Environment 50: 119–128. 10.1016/j.trd.2016.10.030.

[ece370969-bib-0051] Wang, Y. , J. Qu , Y. Han , et al. 2022. “Impacts of Linear Transport Infrastructure on Terrestrial Vertebrate Species and Conservation in China.” Global Ecology and Conservation 38: e02207.

[ece370969-bib-0052] Ważna, A. , A. Kaźmierczak , J. Cichocki , J. Bojarski , and G. Gabryś . 2020. “Use of Underpasses by Animals on a Fenced Expressway in a Suburban Area in Western Poland.” Nature Conservation 39: 1–18.

[ece370969-bib-0053] Whittington, J. , M. Hebblewhite , and R. W. Baron . 2022. “Towns and Trails Drive Carnivore Movement Behaviour, Resource Selection, and Connectivity.” Movement Ecology 10: 17. 35395833 10.1186/s40462-022-00318-5 PMC8994267

[ece370969-bib-0054] Xi, C. , Y. Chi , T. Qian , W. Zhang , and J. Wang . 2020. “Simulation of Human Activity Intensity and Its Influence on Mammal Diversity in Sanjiangyuan National Park, China.” Sustainability 12: 4601.

[ece370969-bib-0055] Xia, L. , Q. Yang , Z. Li , Y. Wu , and Z. Feng . 2007. “The Effect of the Qinghai‐Tibet Railway on the Migration of Tibetan Antelope *Pantholops hodgsonii* in Hoh‐Xil National Nature Reserve, China.” Oryx 41: 352–357.

[ece370969-bib-0056] Xu, W. , Q. Huang , J. Stabach , H. Buho , and P. Leimgruber . 2019. “Railway Underpass Location Affects Migration Distance in Tibetan Antelope (*Pantholops hodgsonii*).” PLoS One 14: e0211798. 30716135 10.1371/journal.pone.0211798 PMC6361455

[ece370969-bib-0057] Xu, W. , X. Li , S. L. Pimm , et al. 2016. “The Effectiveness of the Zoning of China's Protected Areas.” Biological Conservation 204: 231–236.

[ece370969-bib-0058] Yang, Y. , Y. Wang , H. Zhou , X. Chen , S. Tao , and Y. Kong . 2024. “Evaluation of the Effect of Road Barriers on Wildlife Habitats.” Transportation Research Part D: Transport and Environment 131: 104218.

[ece370969-bib-0059] Zhang, C. , W. Ma , C. Chen , M. Wang , W. Xu , and W. Yang . 2022. “Changes of Habitat Pattern for Goitered Gazelle in the Xinjiang Kalamaili Mountain Ungulate Nature Reserve Under the Influence of Major Projects.” Biodiversity Science 30: 21176.

